# Supplementary material for: Effects of the Response to the COVID-19 Pandemic on Assault-Related Head Injury in Melbourne: A Retrospective Study
Source: Int J Environ Res Public Health. 2022 Dec 21;20(1):63. doi: 10.3390/ijerph20010063 (PMC9819794; doi:10.3390/ijerph20010063)
Supplement: Supplementary file 1 [file ijerph-20-00063-s001.zip › Supplementary Table S4.pdf]

Supplemental Table S4. Summary of publications examining the effect of the COVID pandemic on interpersonal violence (according to type of violence and type of data collection)

	Paper	Source	Location	Pandemic period	Type of data	Type of violence	Outcome (pre vs post)
1	Abrams, 2021	Journal article	25 large US cities	May 2020	Jail, incidents, arrests	Domestic violence	↓
1	Abrams, 2021	Journal article	25 large US cities	May 2020	Jail, incidents, arrests	Simple and aggravated assault	↓
2	Ashby, 2020	Journal article	16 large US cities	May 2020	Police-recorded crime data	Serious assaults in public	=
2	Ashby, 2020	Journal article	16 large US cities	May 2020	Police-recorded crime data	Serious assaults in residence	=
3	Balmori de la Miyar et al. 2021	Journal article	Mexico City	Jan-May 2020	Administrative crime data	Assault-battery	↓
3	Balmori de la Miyar et al. 2021	Journal article	Mexico City	Jan-May 2020	Administrative data	Domestic violence	↓
4	Boxall et al. 2020	Institutional report	Australia	May-June 2020	Survey	Domestic violence experienced by women	↑
5	Brink et al. 2021	Journal article	Western and Southern European countries	Within 50 days of start of movement restrictions in 2020	Government websites, news articles and main charity for DV and PV	Domestic, partner or violence against women	↑ in six cities<br>↓ in two cities<br>= in two cities
6	Bullinger et al., 2021	Journal article	Chicago, IL, USA	to Apr 2020	Calls for service to police	Domestic violence (including domestic violence, domestic disturbance, domestic battery, and child abuse)	↑
6	Bullinger et al., 2021	Journal article	Chicago, IL, USA	to Apr 2020	Officer reports and arrests	Domestic crimes	↓
7	Burgess et al. 2021	Institutional report	Victoria, AUS	Apr-Dec 2020	Police crime data	Non-family common assault	↓
7	Burgess et al. 2021	Institutional report	Victoria, AUS	Apr-Dec 2020	Police crime data	Non-family serious assault	↓
7	Burgess et al. 2021	Institutional report	Victoria, AUS	Apr-Dec 2020	Police crime data	Family common assault	↑
7	Burgess et al. 2021	Institutional report	Victoria, AUS	Apr-Dec 2020	Police crime data	Family serious assault	↓
7	Burgess et al. 2021	Institutional report	Victoria, AUS	Apr-Dec 2020	Police crime data	Family violence incidents (family violence incidents, interfamilial-related sexual offences, and child abuse)	↑
8	Campedelli et al. 2020	Journal article	Chicago, IL, USA	Mar-May 2020	Community-wise reported crime incidents from Chicago Data Portal	Assaults	↓ overall<br>↓ in 23% of communities<br>↑ in one community<br>= in the rest
9	Campedelli et al. 2021	Journal article	Los Angeles, CA, USA	March 2020	Crime reports	Assault (battery)	↓
9	Campedelli et al. 2021	Journal article	Los Angeles, CA, USA	March 2020	Crime reports	Assault with a deadly weapon	=
9	Campedelli et al. 2021	Journal article	Los Angeles, CA, USA	March 2020	Crime reports	IPV (including assault)	=

	Paper	Source	Location	Pandemic period	Type of data	Type of violence	Outcome (pre vs post)
10	Di Franco et al. 2020	Journal article	Sicily, Italy	to June 2020	ED admissions	Domestic violence	↓ in absolute numbers but ↑ in women as proportion of total victims
11	Evans et al. 2021	Journal article	Atlanta, GA, USA	to week 31 of 2020	Police crime data	Domestic crimes	↑
12	Gerell et al. 2020	Journal article	Sweden	to week 21 of 2020	Police data	Outdoor assault	↓
12	Gerell et al. 2020	Journal article	Sweden	to week 21 of 2020	Police data	Domestic violence (indoor assault)	↓
13	Gosangi et al. 2021	Journal article	North-eastern USA	to May 2020	Administrative health records	IPV	↓ IPV (total, incl. physical and nonphysical) ↑ physical IPV and severity
14	Hsu and Henke, 2021	Journal article	35 cities and 1 county, USA	Mar-May 2020	Police incidents, calls for service	Domestic violence (includes reports of domestic assault, domestic battery, or a family fight)	↑
15	Lausi et al. 2021	Systematic review	Worldwide	1st half of 2020	Help professionals	Intimate Partner Violence (IPV)	↓ physical assault but ↑ severity ≈ or ↓ IPV according to police/healthcare data
15	Lausi et al. 2021	Systematic review	Worldwide	1st half of 2020	Victims - On-line survey	Intimate Partner Violence (IPV)	↑ IPV non-physical episodes ↓ assaults but ↑ severity
16	Leslie and Wilson 2020	Journal article	14 US cities	Mar-May 2020	Police calls for service	Domestic violence	↑ first five weeks spike
17	McLay et al. 2021	Journal article	Chicago, IL, USA	March 2020	Police reports	Domestic violence (exclusively reports that involved physical or sexual violence)	↓
18	McCrary and Sanga 2020	Journal article	14 US cities	Jan-Apr 2020	Police calls for service and mobile device location data	Domestic violence	↑
19	Mohler et al., 2020	Journal article	2 US cities	Jan-Apr 2020	Police calls for service	Assaults	=
19	Mohler et al., 2020	Journal article	2 US cities	Jan-Apr 2020	Police calls for service	Domestic violence	↑
20	Moslehi et al. 2021	Journal article	NSW and VIC, AUS	to Dec 2020	Monthly count of reported offences	Domestic assault	↓
20	Moslehi et al. 2021	Journal article	NSW and VIC, AUS	to Dec 2020	Monthly count of reported offences	Other assault	↓

	Paper	Source	Location	Pandemic period	Type of data	Type of violence	Outcome (pre vs post)
21	Muldoon et el., 2021	Journal article	Ottawa, Canada	Mar-May 2020	ED admissions	Domestic assault	↓
22	Nivette et al. 2021	Journal article	27 cities across 23 countries in the Americas, Europe, the Middle East and Asia	1 month before to 4 months after start of stay-at-home orders	Crime data	Assault	↓
23	Nix & Richards 2021	Journal article	6 US cities	to Dec 2020	Police calls for service	Domestic violence	↑ first few weeks spike
24	Payne et al. 2020	Journal article	Queensland, AUS	Mar-Apr 2020	Offence rates	Common assault	↓
24	Payne et al. 2020	Journal article	Queensland, AUS	Mar-Apr 2020	Offence rates	Serious assault	↓
24	Payne et al. 2020	Journal article	Queensland, AUS	Mar-Apr 2020	Breach of orders	Domestic violence	=
25	Perez-Vincent et al. 2020	Institutional technical note	Buenos Aires, Argentina	to Apr 2020	Calls to hotline	Domestic violence	↑
26	Pfitzner et al. 2020	Institutional report	Victoria, AUS	Apr-May 2020	Survey of practitioners responding to women experiencing violence	Violence against women	↑
27	Piquero et al. 2020	Journal article	Dallas, TX, USA	Mar-Apr 2020	Family Violence Incident List - Crime data	Domestic violence (including misdemeanour, felony, child, elderly, and sexual assault. and family violence incidents)	↑ first two weeks spike
28	Piquero et al. 2021	Systematic review	Worldwide (18 studies): US cities, Mexico, Italy, Sweden, Australia, Argentina, India	Weeks-months from start of restrictions	Police crime data, crime reports, police calls for service, ED records, calls to hotline, etc.	Domestic violence, intimate partner violence, violence against women	↑ overall<br>↑ 29 of study estimates reported<br>↓ eight of study estimates reported<br><br>Effect size analysis<br>↑ 12 studies<br>= four studies<br>↓ one study
29	Ravindran & Shah 2021	Institutional working paper	India	to May 2020	Records of complaints by the National Commission for Women	Domestic violence	↑
30	Rhodes et al. 2020	Journal article	South Carolina, USA	to April 2020	ED records	Domestic violence (assault)	↑
31	Victorian Injury Surveillance Unit 2021	Institutional report	Victoria, AUS	to December 2020	ED records	Assault-related injuries at home	↑ in both males and females

Key to symbols: ↑ increase, ↓ decrease, **=** no change

Type of data and type of violence are specified as originally described in the source paper
