# Supplementary material for: Effects of the Response to the COVID-19 Pandemic on Assault-Related Head Injury in Melbourne: A Retrospective Study
Source: Int J Environ Res Public Health. 2022 Dec 21;20(1):63. doi: 10.3390/ijerph20010063 (PMC9819794; doi:10.3390/ijerph20010063)
Supplement: Supplementary file 1 [file ijerph-20-00063-s001.zip › Supplementary Tables1-3.pdf]

Supplementary Tables

Supplementary Table 1. *Who* was a victim of assault-related TBI in Melbourne, Australia, between July 30th of 2018 and October 30th of 2021?

Sample characteristics	Total<br>n (%)	Assault Subgroups		
		Random<br>n (%)	Domestic<br>n (%)	Prison<br>n (%)
**All**	1232	900 (73.1)	111 (9.0)	221(17.9)
**Sex**				
Male	1016 (82.5)	776 (86.2)	29 (26.1)	211 (95.5)
Female	216 (17.5)	124 (13.8)	82 (73.9)	10 (4.5)
**Age**				
18-39	842 (68.3)	605 (67.2)	73 (65.8)	164 (74.2)
40-59	345 (28)	261 (29)	30 (27)	54 (24.4)
60+	45 (3.7)	34 (3.8)	8 (7.2)	3 (1.4)
**Alcohol Consumption**				
No	672 (54.5)	385 (42.8)	69 (62.2)	218 (98.6)
Yes	325 (26.4)	308 (34.2)	17 (15.3)	0 (0)
Unknown	235 (19.1)	207 (23)	25 (22.5)	3 (1.4)
**Nationality**				
Australian	752 (61)	535 (59.4)	70 (63.1)	147 (66.5)
Indigenous[a]	76 (10.2)	55 (10.4)	7 (10)	14 (9.7)
Non-Australians	419 (34)	348 (38.7)	40 (36)	31 (14)
Unknown	61 (5)	17 (1.9)	1 (0.9)	43 (19.5)

[a] Aboriginal & Torres Strait Islanders, Percentages are based on Australian group rather than total group.

Supplementary Table 2. *Where* were victims of assault-related TBI in Melbourne, Australia, attacked between July 30th of 2018 and October 30th of 2021?

**Location of Injury**	n (%)
Home	175 (14.2)
Road/Street/Highway	295 (23.9)
Place for Recreation	88 (7.1)
Workplace and other public places	234 (18.9)
Prison	221 (17.9)
Unknown	222 (18.0)

Supplementary Table 3. *When* did victims of assault-related TBI in Melbourne, Australia, present at the ED between July 30th of 2018 and October 30th of 2021?

	**All**	**Random**	**Domestic**	**Prison**
**ED Presentation Time**				
Morning (6:00-12:00)	189 (15.3)	151 (16.8)	20 (18.0)	18 (8.4)
Afternoon (12:00-18:00)	343 (27.8)	227 (25.2)	29 (26.1)	87 (39.4)
Evening (18:00-24:00)	392 (31.8)	249 (27.7)	36 (32.4)	107 (48.4)
Night (0:00-6:00)	308 (25.0)	273 (30.3)	26 (23.4)	9 (4.1)
**TOI Reporting >24 Hours**				
No	617 (50.08)	433 (48.1)	48 (43.2)	136 (61.5)
Yes	104 (8.4)	80 (8.9)	12 (10.8)	12 (5.4)
Unknown	511 (41.5)	387 (43.0)	51 (46.0)	73 (33.0)

ED, emergency department; TOI, time of injury
